# Influenza activity and regional mortality for non-small cell lung cancer

**DOI:** 10.1038/s41598-023-47173-x

**Published:** 2023-12-07

**Authors:** Connor J. Kinslow, Yuankun Wang, Yi Liu, Konstantin M. Zuev, Kunal R. Chaudhary, Tony J. C. Wang, Ciro Donalek, Michael Amori, Simon K. Cheng

**Affiliations:** 1 Department of Radiation Oncology, Vagelos College of Physicians and Surgeons, Columbia University Irving Medical Center, 622 West 168th Street, BNH B011, New York, NY 10032 USA; 2 Virtualitics Inc, 225 S. Lake Avenue Suite 120, Pasadena, CA 91101 USA; 3 California Institute of Technology, 1200 E. California Blvd., Pasadena, CA 91125 USA; 4 Herbert Irving Comprehensive Cancer Center, Vagelos College of Physicians and Surgeons, Columbia University Irving Medical Center, 1130 St Nicholas Ave, New York, NY 10032 USA

**Keywords:** Cancer epidemiology, Non-small-cell lung cancer, Cancer

## Abstract

Lung cancer is the leading cause of cancer deaths in the United States and worldwide. While influenza illness is known to be particularly dangerous for frail and elderly patients, the relationship between influenza illness and outcomes in patients with cancer remains largely unknown. The Surveillance, Epidemiology, and End Results (SEER) database was queried to identify patients with non-small cell lung cancer (NSCLC) diagnosed between 2009 and 2015. Influenza-like illness (ILI) activity, provided by the Outpatient Influenza-like Illness Surveillance Network of the Center of Disease for Control and Prevention, was merged with the SEER dataset on the state-month level. Regional monthly mortality rates were compared during low versus high flu months in this ecological cohort study. 202,485 patients with NSCLC from 13 SEER-reporting states were included in the analysis. 53 of 1049 state-months (5.1%) had high flu activity. Monthly mortality rates during low and high flu months were 0.041 (95% CI 0.041–0.042) and 0.051 (95% CI 0.050–0.053), respectively (RR 1.24 [95% CI 1.21–1.27]). The association between ILI activity and mortality was observed at the individual state level and in all clinical and regional subgroups. Increased regional influenza activity is associated with higher mortality rates for patients with NSCLC. Vaccine-directed initiatives and increased awareness amongst providers will be necessary to address the growing but potentially preventable burden of influenza-related lung cancer deaths in the U.S.

## Introduction

Each year, influenza affects approximately 15% of the U.S. population, leading to more than 334,000 hospitalizations and 41,000 deaths, with $26.7 billion direct and indirect associated costs^1^. Though influenza is known to be particularly devastating for frail and elderly patients^2, 3^, data to support vulnerability in patients with cancer is much less robust. Patients with cancer are often immunocompromised due to treatment with chemotherapy or their underlying disease and, therefore, more susceptible to microbial infections. Patients with cancer are assumed to be at high risk of influenza-related morbidity and mortality^4^, but the majority of data pertaining to influenza-related outcomes is derived from older and smaller retrospective case series^5–11^.

The Advisory Committee on Immunization Practice (ACIP) and the American Society for Clinical Oncology (ASCO) recommend annual influenza vaccination for all individuals, including those with cancer or those receiving chemotherapy^4, 12^. However, it is recognized that there is a lack of level 1 and retrospective evidence to support this recommendation^12, 13^. Influenza vaccination rates remain low amongst patients with cancer and their family members in the U.S. and worldwide, largely due to an absence of recommendations by individual treating providers^1, 14–16^. Reasons for the lack of provider initiative include a lack of awareness of the seriousness of influenza infection in patients with cancer and a lack of professional guidelines or awareness of professional guidelines^17–19^. Therefore, a better understanding of the impact of influenza on the outcomes of patients with cancer would allow researchers and physicians to better access risks of exposure and the potential benefits of vaccination. This may have the effect of increasing vaccination rates, both by increasing awareness amongst treating physicians and by bolstering the strength of evidence behind recommendations from professional societies. The burden of influenza-related morbidity and mortality in patients with cancer is expected to rise, consequent to an aging population and an increase in cancer prevalence^20^. It is, therefore, important to address this growing and potentially preventable problem.

In this ecological study, we explore the relationship between regional influenza activity and non-small cell lung cancer (NSCLC) mortality rates across several flu seasons in the United States. We hypothesized that patients with lung cancer would be susceptible to influenza-related mortality, given that both disease processes have pulmonary tropism. We used population-level data on influenza-like illness (ILI) provided by the Center for Disease Control and Prevention (CDC) and non-small cell lung cancer (NSCLC) mortality provided by the National Cancer Institute’s (NCI) Surveillance, Epidemiology, and End Results (SEER) Program. Combining these two datasets, we were able to achieve spatiotemporal resolution at the state-month level.

## Methods

### Data sources

The SEER Program is the NCI’s authoritative source for population-based cancer incidence and survival in the U.S.^21^. It is also considered the gold standard for cancer data collection internationally^22^. Data is populated from national cancer registries in 13 contributing states and encompasses approximately 34.6% of the U.S. population^23–25^. Mortality data reported to SEER is provided by the National Center for Health Statistics. The SEER Program is updated annually for follow-up on vital status and routinely undergoes quality-control checks. Data were collected and analyzed as previously reported^26–34^.

FluView Interactive is a dashboard produced by the Epidemiology and Prevention Branch in the Influenza Division at the CDC^35^. The U.S. Outpatient Influenza-like Illness Surveillance Network (ILINet) consists of more than 3500 providers in 50 states who report more than 47 million patient visits per year.

This study was exempt from review by the Columbia University Institutional Review Board.

### Sample selection and coding

The SEER database was queried (November, 2017 submission, including data from 1973 to 2015)^36^ to identify all cases of NSCLC^37^ within the lung and bronchus, diagnosed between October 1, 2008 and December 31, 2015. AJCC 6th Edition Staging was the most modern staging system that was uniformly available for all patients^38, 39^. Percent of persons below the poverty level, median household income, normalized cost of living index, and rural urban continuum are recorded at the county level in which the individual patient resides. Cases diagnosed at autopsy or that could have 0 days of follow-up, cases with prior malignancies, and cases with unknown AJCC Staging were excluded^40^.

ILI activity level is provided at the state level for each week of the year. Weekly ILI activity levels were averaged during each month. The CDC and SEER datasets were then merged at the state-month level.

### Primary measurements and outcomes

ILI is defined as a fever (temperature of 100 °F [37.8 °C] or greater) and a cough and/or sore throat without a known cause other than influenza. ILI activity is calculated based on the regional percentage of patient visits for ILI reported during each week. Activity levels compare the mean reported percent of ILI visits for a given week with the mean reported percent of visits during non-influenza weeks. ILI activity levels range from 1 to 10, with an activity level of 1 corresponding to values below the mean, 2 corresponding to values within one standard deviation of the mean, and each level above 2 corresponding to an additional standard deviations above the mean. Activity levels of 8–10 are considered high (hereafter referred to as high flu months). Overall mortality rate was defined as the number of patients with NSCLC who died of any cause within a given month, divided by the total number of patients at risk of death. One-month mortality rate was defined as the number of patients who were newly diagnosed with NSCLC and died of any cause within a given calendar month, divided by the total number of patients who were newly diagnosed and at risk of death during that same month.

### Statistical analysis

In this ecological study, bootstrapping^41^ was used to determine the distributions of both overall and one-month mortality rates. For each state and month, a sample was drawn—with replacement—from the raw mortality data, with the number of samples equal to the number of cases in the state in that month. A sample mortality rate was then calculated using the data across all months and states, for both low and high flu groups. This process was then repeated 10,000 times in order to determine the distribution of mortality rate. The 95% confidence intervals (95% CI) for the mortality rates were determined by taking the middle 95% of the sampled mortality rates. To calculate the relative risk (RR) and its 95% CI, the sampled mortality rates for the high flu group was divided by the sampled mortality rate of the low flu group. In addition, the 95% CI of the low flu group was determined by dividing the mortality rate from 10,000 samples of the high flu group by an additional 10,000 samples of mortality rate from the low flu group. All statistical analyses were conducted using Python Version 3.5.5 (Python Software Foundation, Delaware, United States) and the NumPy module^42^.

## Results

### Patient selection and characteristics

Our initial query identified 282,795 patients with a diagnosis of NSCLC (Supplemental Fig. 1). After applying our exclusion criteria, there were 202,485 cases remaining. Median follow-up and survival times were 8 and 11 months, respectively, with 141,651 deaths. Pneumonia and influenza was listed as the cause of death in 0.4% (*n* = 592) of all death certificates (Supplemental Table 1). Demographical and clinical features of patients are displayed in Table 1. The majority of patients lived in metropolitan areas (85.5%) with greater than 1,000,000 people (57.2%). California and Georgia accounted for the largest proportion of patients (33.4 and 12.7%, respectively), while fewer patient records were collected from Alaska (274 [0.1%]), Utah (2373 [1.2%]), Hawaii (3113 [1.5%]), and New Mexico (3487 [1.7%]).

**Table 1:** Patient demographical and clinical characteristics.

	Count	%
Age		
0–65	78,316	38.7
65+	124,169	61.3
Sex		
Female	95,146	47.0
Male	107,339	53.0
Race		
American Indian/Alaska Native	1041	0.5
Asian or Pacific Islander	14,672	7.2
Black	25,013	12.4
White	161,346	79.7
Unknown	413	0.2
AJCC 6th stage		
I	44,477	22.0
II	9329	4.6
III	49,841	24.6
IV	96,769	47.8
Occult	2069	1.0
Cancer-directed surgery		
No surgery	154,732	76.4
Surgery	46,754	23.1
Unknown	999	0.5
Population size		
< 250,000	46,065	22.7
250,000–1,000,000	40,329	19.9
Greater than 1,000,000	115,801	57.2
Unknown	290	0.1
Population type		
Rural	4017	2.0
Urban	25,096	12.4
Metropolitan	173,082	85.5
Unknown/other	290	0.1
State		
Alaska	274	0.1
California	68,239	33.7
Connecticut	10,282	5.1
Georgia	25,760	12.7
Hawaii	3113	1.5
Iowa	9057	4.5
Kentucky	18,703	9.2
Louisiana	14,512	7.2
Michigan	13,035	6.4
New Jersey	22,686	11.2
New Mexico	3487	1.7
Utah	2373	1.2
Washington	10,964	5.4

### Distribution of high flu months

**Figure 1:**
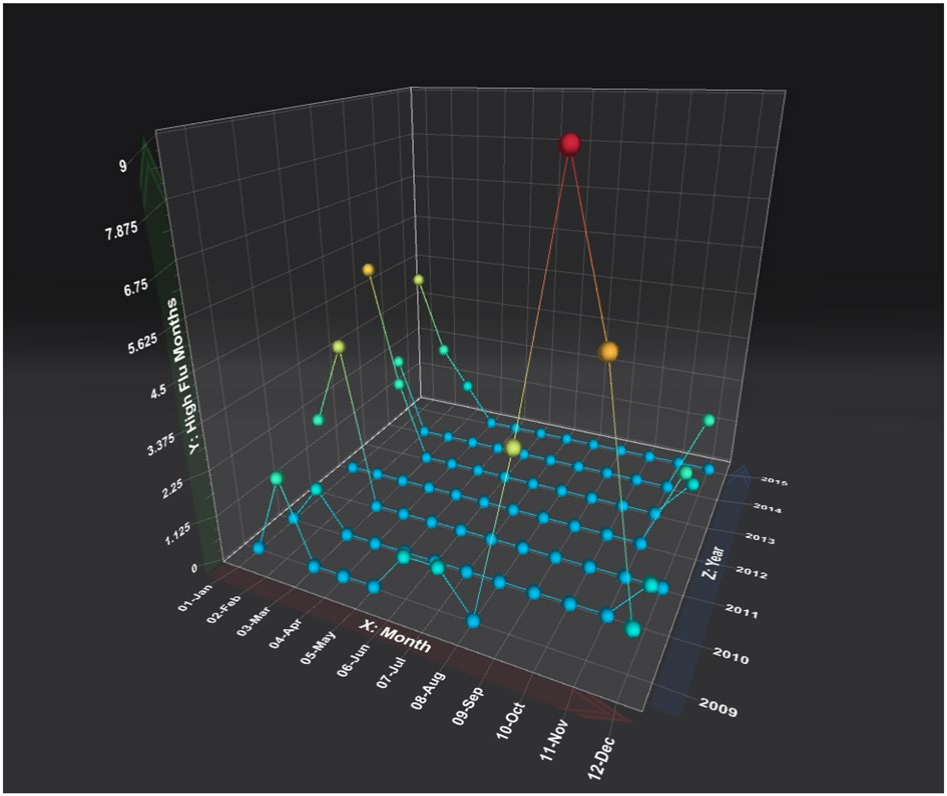
Distribution of high flu months over the study period. Y-axis corresponds to the total number of states with high ILI activity during a given month and year.

1041 state-months were observed from the 13 SEER-reporting states, 53 (5.1%) of which were considered high flu months. The distribution of high flu months throughout the study period is illustrated in Fig. 1. 2009 contained the highest proportion of high flu months (24/52), followed by 2013 (8/52). High activity flu months generally occurred between October and February. Louisiana had the highest proportion of high flu months (18.8%), followed by Georgia (12.0%, Table 2).

**Table 2:** Distribution of high flu months by state.

State	Low flu months	High flu months	% high flu months
Alaska	78	2	2.6
California	79	2	2.5
Connecticut	72	2	2.8
Georgia	75	9	12.0
Hawaii	78	4	5.1
Iowa	77	1	1.3
Kentucky	78	2	2.6
Louisiana	69	13	18.8
Michigan	82	1	1.2
New Jersey	78	4	5.1
New Mexico	74	5	6.8
Utah	77	6	7.8
Washington	71	2	2.8

### Influenza activity and mortality rate

**Figure 2:**
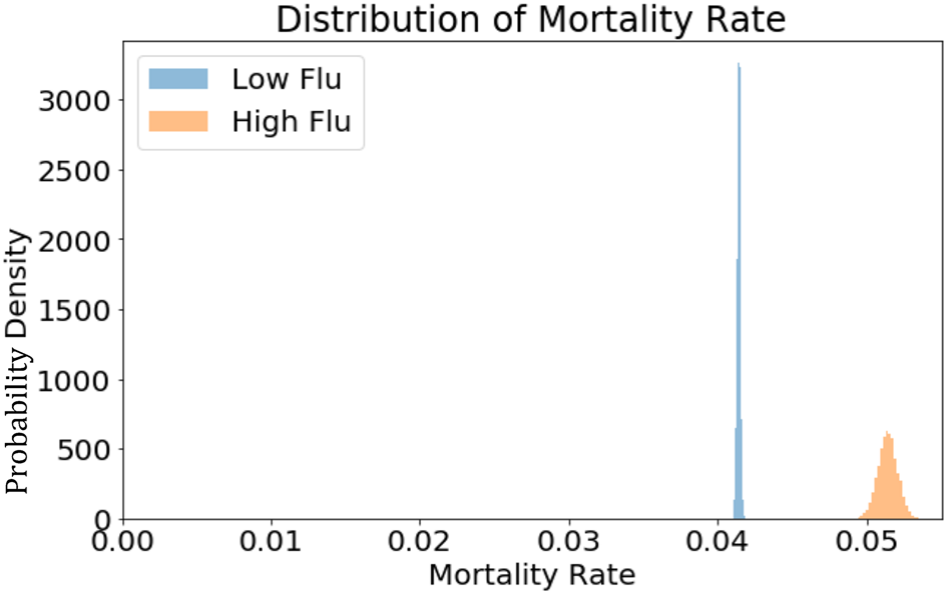
Overall monthly mortality rates during low and high flu months.

**Figure 3:**
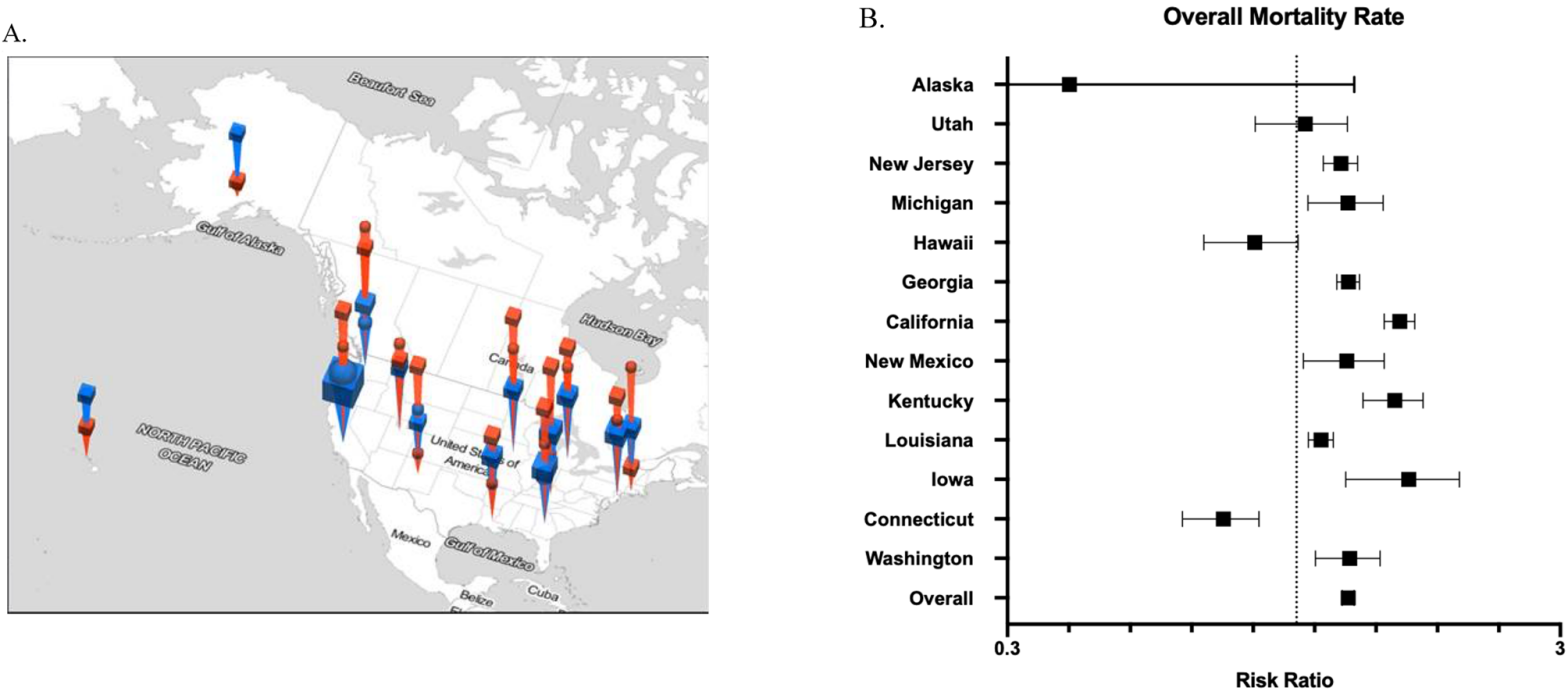
Risk ratio for overall mortality rate during high flu months (**A**, **B**). (**A**) Map of SEER-reporting states in the U.S. Low flu months are represented by blue bars. High flu months are represented by red bars. Squares and circles represent risk ratio of overall and one-month (see methods) mortality rates, respectively. Height corresponds to RR of mortality during high vs. low flu months. Width of bars corresponds to the number of cases available for analysis. (**B**) Dotted line intersects x-axis at one. Error bars represent 95% confidence interval.

The overall monthly mortality rate for all patients was 0.042 deaths per person at risk. The Supplemental Video shows a time-lapsed map of the United States with ILI activity and mortality rates for each SEER-reporting state. During low and high flu months, the monthly mortality rates were 0.041 (95% CI 0.041–0.042) and 0.051 (95% CI 0.050–0.053), respectively (RR 1.24 [95% CI 1.21–1.27], Fig. 2). To account for regional differences in patient characteristics and mortality rates^43^, we examined the relationship between influenza activity and NSCLC mortality at the individual state level (Fig. 3). In 9 out of 13 states, there was a statistically significant association between influenza activity and mortality rate, versus 1 state (Connecticut) in which the mortality rate during high flu months was significantly lower. In the states with the largest populations (California and Georgia), the RR for mortality during high versus low flu months were 1.54 (95% CI 1.44–1.64) and 1.24 (95% CI 1.18–1.30), respectively.

**Figure 4:**
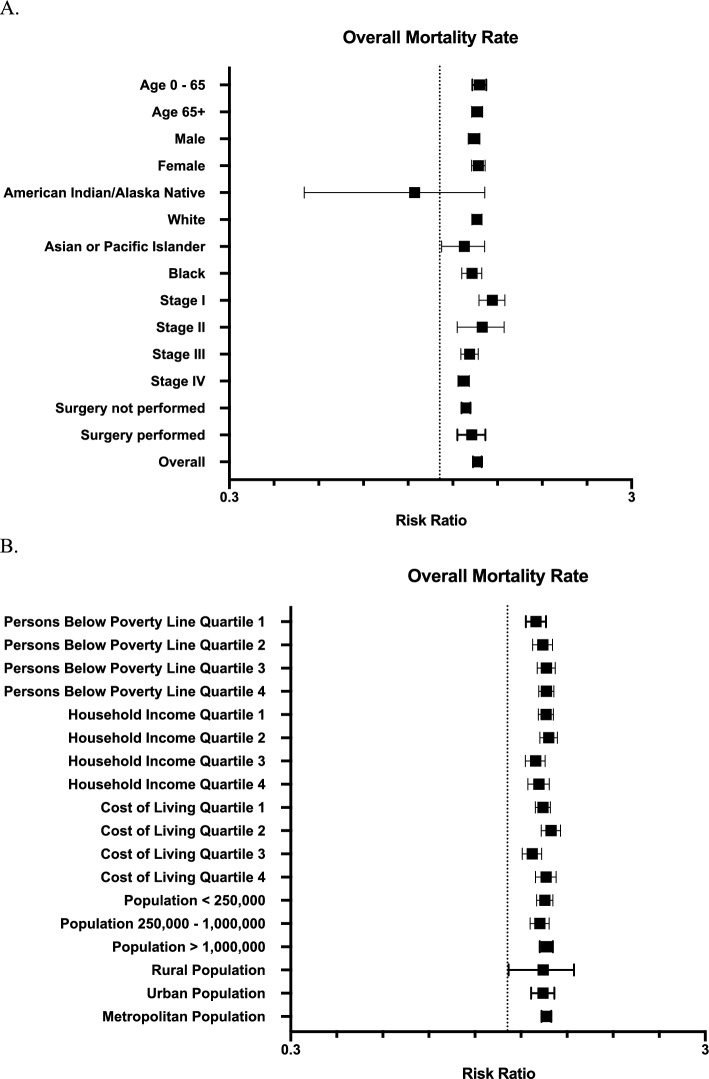
Risk ratio for overall mortality rate during high flu months in subgroups stratified by individual patient (**A**) and regional (**B**) clinical and demographical features. Dotted line intersects x-axis at one. Error bars represent 95% confidence interval.

We further examined the relationship between influenza activity and mortality in subgroups based on clinical and regional factors (Fig. 4). In all clinical subgroups, there was a significantly higher mortality rate during high flu months (Fig. 4A), with the exception of American Indian/Alaska Natives, for which there were exceptionally few cases available (0.5% of total population). There was also a significantly higher mortality rate in all regional subgroups (Fig. 4B). The RR for mortality during high versus low flu months increased incrementally based on the percentage of persons below the poverty line. The RR for mortality was 1.17 (95% CI 1.11–1.24), 1.22 (95% CI 1.15–1.28), 1.24 (95% CI 1.18–1.30), and 1.24 (95% CI 1.19–1.29) for Quartiles 1, 2, 3, and 4, respectively.

### Sensitivity analysis

The majority of high flu month occurred during winter months (Fig. 1). To rule out that the association between influenza activity and mortality was due to a general increase in mortality during winter months, and not specific to influenza, we analyzed the relationship between influenza activity and mortality in winter and non-winter months separately. During winter and non-winter months, the RRs for mortality during high versus low flu months were 1.07 (95% CI 1.03–1.10) and 1.61 (95% CI 1.54–1.67), respectively (Supplemental Fig. 2).

To minimize the influence of time on the outcome of interest, we performed a sensitivity analysis for the secondary outcome of one-month mortality. The 1-month mortality rates during low and high flu months were 0.094 (95% CI 0.093–0.095) and 0.102 (95% CI 0.096–0.109) deaths per persons diagnosed, respectively, with an RR of 1.08 (95% CI 1.03–1.13) during high flu months (Supplemental Fig. 3). We further examined the relationship between influenza activity and one-month mortality during winter and non-winter months (Supplemental Fig. 4), at the state level, and after stratifying by clinical and regional subgroups, as described previously (Supplemental Fig. 5). Qualitatively, our main findings were not substantially changed. However, there was no longer an incremental increase in the RR of mortality during high flu months as the percentage of persons below the poverty line increased.

### Ethical statement

This study was exempt from review by the Columbia University Institutional Review Board.

### Conference presentation

A preliminary version of this study was presented at the American Society of Clinical Oncology Annual Meeting 2019 (May 31–June 4, 2019, Chicago, Illinois) as an abstract.

## Discussion

In this ecological study, we found that regional NSCLC mortality rates in the United States were higher during months with high ILI activity. This relationship was observed at the individual state-level and in all clinical and regional subgroups. We found an incremental increase in the relative risk of mortality with increasing percentages of patients below the poverty line. This may be due to lower vaccination rates in lower income communities^1, 15, 44^.

Previous research has found negligible fluctuations in seasonal mortality rates for patients with lung cancer or other malignancies^45, 46^. Although influenza seasons generally occur between November and March, our ability to detect differences in mortality rates during high and low flu months is likely due to the spatiotemporal resolution of our study at the state-month level.

Several older, smaller retrospective case series have suggested that influenza frequency and morbidity is higher in patients with cancer, though the majority of these studies have focused on hematological malignancies^5, 6^. There are fewer studies that report influenza-related outcomes in patients with solid malignancies and data suggests that outcomes are better for patients with solid cancers^7, 8^. To our knowledge, there is only one population-based study that examines influenza-related cancer outcomes^7^. Using data from the National Inpatient Sample, Cooksley et al. found that patients with cancer who were hospitalized for influenza-related illness have a longer length of stay, higher cost of hospitalization, and higher mortality rate than that of the general population. The mortality rate for hospitalized cancer patients was 9%. Several other studies, including one large multicenter retrospective and one large prospective study, have reported similar mortality rates in hospitalized cancer patients^9–11^. Among patients with cancer, the mortality rate was highest for those with lung cancer, reaching 12.4%. The study concluded that patients with cancer that were hospitalized with influenza-related infections are 10 times more likely to die than the general population.

By assuming that all excess deaths that occurred during high flu months were due to influenza infection, we can approximate that 1.2% of deaths in our cohort were attributable to influenza infections. By comparison, pneumonia and influenza was listed as the cause of death in 0.4% of patients in our cohort, based on death certificate records. This discrepancy is expected, as it is known that influenza-related deaths, based solely on death certificate records, is a gross underestimation of the seasonal influenza’s true impact^47, 48^.

Two studies, one retrospective and one prospective, have shown reduced influenza and pneumonia diagnoses, chemotherapy interruptions, and mortality in patients with solid malignancies who are vaccinated^9, 15^. A Cochran Systematic Review concluded that the benefits outweigh the potential risks when vaccinating adults with cancer against influenza^13^. Additionally, two studies have demonstrated cost-effectiveness of vaccination in patients with cancer based on analytical modeling^49, 50^. A U.S. study concluded that influenza vaccination is cost-effective for working-age cancer patients with a life expectancy greater than 2.8 months^50^. Given that the median survival times for all stages and stage IV patients with NSCLC are 11 and 4 months, respectively^51^, it appears that vaccination would be a cost-effective strategy in any working-age patient with NSCLC.

Despite recommendations from professional societies to vaccinate patients with cancer annually, vaccination rates remain low in the U.S. and around the world^9, 14–16, 52^. In the U.S., only 40% of elderly patients with colorectal cancer received influenza vaccination^15^. Several studies have shown that the main reason for absence of vaccination in patients with cancer is a lack of incitation by the treating physician^16, 18^. Virtually every study that examined vaccination rates in patients with cancer concluded that increased awareness amongst practitioners was necessary to improve vaccination rates^14, 16, 18, 19, 52^.

Advantages of the methodology used in this study include the use of large datasets with a population-based approach, representing 34.6% of U.S. patients with cancer. Additionally, it is the first study to assess regional influenza activity and lung cancer mortality over several influenza seasons. The CDC and the SEER program are the two most robust surveillance systems in the U.S. for influenza outbreaks and cancer mortality statistics, respectively. SEER is considered the international gold-standard for population research when measuring cancer incidence and mortality. Mortality is recorded from death certificates, which are linked to individual patient records via their social security numbers. Therefore, our primary outcome should be highly reliable.

### Limitations

A greater proportion of high flu months occurred during 2009, corresponding to the H1N1 pandemic. Because patients at risk of death during the 2009 pandemic would have shorter follow-up times, they may have had higher mortality rates, irrespective of influenza activity. To account for this, we also analyzed the one-month mortality rates.

Although the ILI activity metric is based on an expansive surveillance system, it is known that less than half of patients with influenza symptoms present to their providers. Furthermore, ILI activity is based on presenting symptoms, not laboratory-confirmed influenza, which is the gold-standard for diagnosis. Some ILI visits may have been caused by other respiratory viruses^53^.

Variations in the effect of ILI by state were estimated based on a limited number of observations and should be interpreted with caution. Additionally, differences in ecological conditions, such as patient populations, local policies, access to care, outbreak dynamics, and other confounders may affect the outcome.

## Conclusions

Regional ILI activity is associated with higher mortality rates for NSCLC patients in the U.S. This is the first population-based study to estimate the effects of regional influenza activity on mortality rates in patients with lung cancer over multiple flu seasons. Limitations notwithstanding, our study addresses the sparsity of data on influenza-related outcomes in patients with cancer. These findings may be used to: (1) Bolster evidence supporting professional guidelines for annual influenza vaccination in patients with cancer, (2) Estimate influenza-related mortality in patients with lung cancer in the U.S., (3) Project mortality in upcoming flu seasons based on predicted influenza activity, (4) Estimate cost-effectiveness of influenza vaccination in patients with lung cancer. Influenza is a major source of cancer-associated morbidity and mortality in the U.S. Vaccine-directed initiatives and increased awareness amongst providers will be necessary to address the growing but potentially preventable burden of influenza-related cancer deaths.

### Supplementary Information

Supplementary Legends.

Supplementary Figures.

Supplementary Table 1.

Supplementary Video 1.

## Data Availability

Data is available upon request to the National Cancer Institute and Center for Disease Control, but restrictions apply to the availability of these data, which were used under license for the current study, and so are not publicly available. Permissions can be obtained through the SEER website (https://seer.cancer.gov/data/access.html).
