# Supplementary material for: Influenza activity and regional mortality for non-small cell lung cancer
Source: Sci Rep. 2023 Dec 7;13:21674. doi: 10.1038/s41598-023-47173-x (PMC10709588; doi:10.1038/s41598-023-47173-x)
Supplement: Supplementary file 1 — Supplementary Legends. [file 41598_2023_47173_MOESM1_ESM.docx]

Supplemental Video- Time-lapsed video of ILI severity and mortality rates in SEER-reporting states during the study period. Each frame represents a new month of the study in chronological order. Height of bars represent ILI activity level. Color of bars correspond to overall monthly mortality rates during a particular month, with cooler colors (blue) representing to lower mortality rates and hotter colors (red) representing higher mortality rates.
