# Supplementary material for: Influenza activity and regional mortality for non-small cell lung cancer
Source: Sci Rep. 2023 Dec 7;13:21674. doi: 10.1038/s41598-023-47173-x (PMC10709588; doi:10.1038/s41598-023-47173-x)
Supplement: Supplementary file 2 — Supplementary Figures. [file 41598_2023_47173_MOESM2_ESM.docx]

Supplemental Figure 1- Patient Selection

Diagnosed 10/2008 – 12/2015: Assessed for eligibility (n = 282,795)

Primary NSCLC of the lung.

Total excluded in Step 1 (n = 2,254)

1. Diagnosed at autopsy
2. Could have zero days of follow-up

Total excluded in Step 2 (n = 69,504)

Prior malignancies

Total included after Step 3 (n = 202,485)

Total excluded in Step 3 (n = 8,552) Unknown AJCC Staging


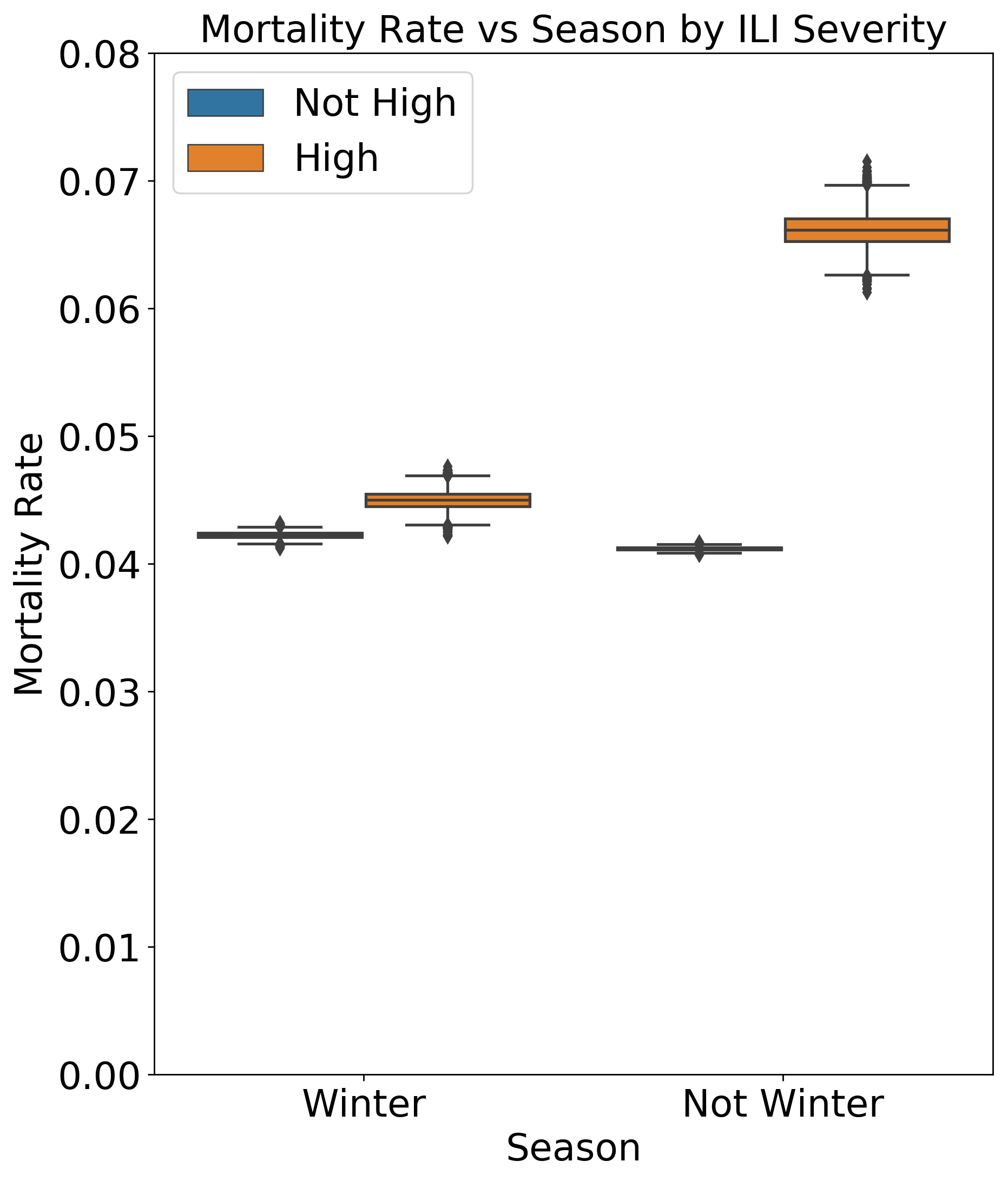


Supplemental Figure 2- Overall monthly mortality rates during winter (December, January, February) and non-winter months. Boxes bound interquartile range. Error bars bound 95% confidence interval.


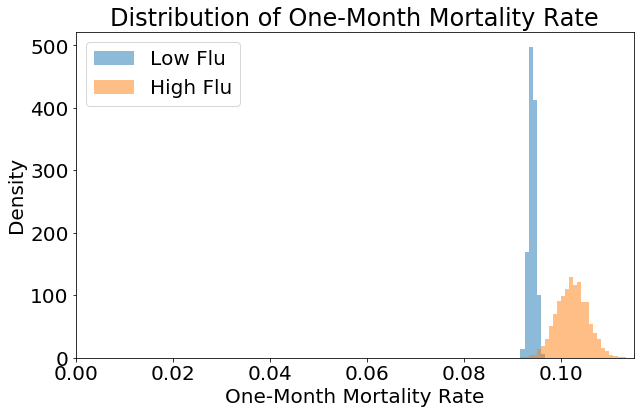


Probability

Supplemental Figure 3- One-monthly mortality rates during low and high flu months.

*
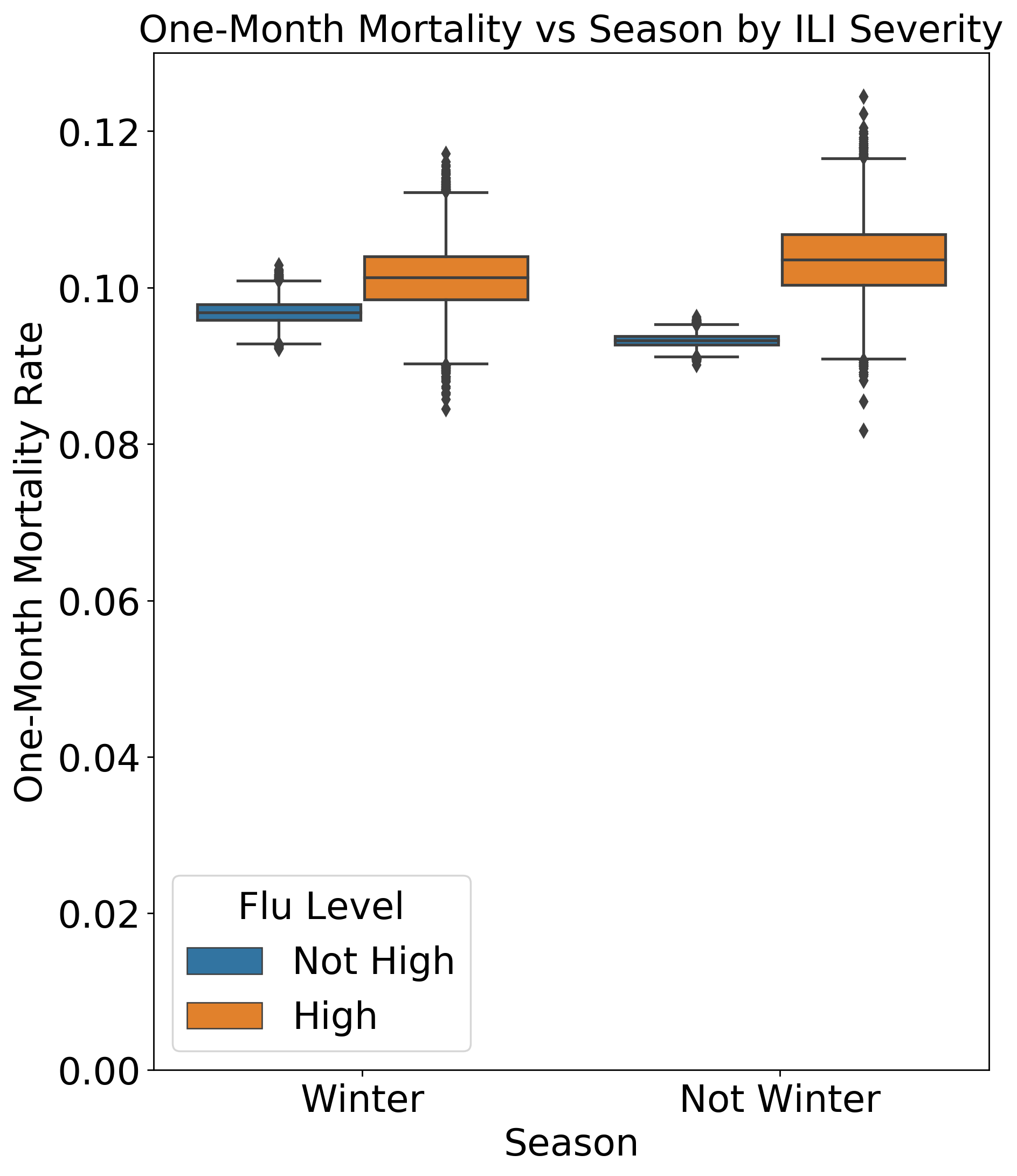
*

Supplemental Figure 4- One-month mortality rates for newly diagnosed cases during winter (December, January, February) and non-winter months. Boxes bound interquartile range. Error bars bound 95% confidence interval.

A.

B.

C.

Supplemental Figure 5- Risk ratio for one-month mortality rate during high flu months in subgroups stratified by state (A) and by individual patient (B) and regional (C) clinical and demographical features. Dotted line intersects x-axis at one. Error bars represent 95% confidence interval.
