# Supplementary material for: Influenza activity and regional mortality for non-small cell lung cancer
Source: Sci Rep. 2023 Dec 7;13:21674. doi: 10.1038/s41598-023-47173-x (PMC10709588; doi:10.1038/s41598-023-47173-x)
Supplement: Supplementary file 3 — Supplementary Table 1. [file 41598_2023_47173_MOESM3_ESM.docx]

Supplemental Table 1- Causes of Death in Patient Population

| Causes of death | *n* (%) |  |
| --- | --- | --- |
| Dead (attributable to this cancer diagnosis) | 121,376 (85.7%) | |
| Lung and Bronchus | 117,808 (83.2%) | |
| Diseases of Heart | 4,533 (3.2%) | |
| Miscellaneous Malignant Cancer | 4,148 (2.9%) | |
| Chronic Obstructive Pulmonary Disease and Allied Condition | 3,194 (2.3%) | |
| Other Cause of Death | 2,533 (1.8%) | |
| State DC not available or state DC available but no COD | 1,797 (1.3%) | |
| Cerebrovascular Diseases | 860 (0.6%) | |
| Pneumonia and Influenza | 592 (0.4%) | |
| All other (combined) | 6,186 (4.4%) | |
| Dead of other cause | 15,652 (11.0%) | |
| Diseases of Heart | 4,437 (28.3%) | |
| Chronic Obstructive Pulmonary Disease and Allied Conditions | 3,120 (19.9%) | |
| Other Cause of Death | 2,467 (15.8%) | |
| Cerebrovascular Diseases | 842 (5.45) | |
| Pneumonia and Influenza | 582 (3.7%) | |
| Accidents and Adverse Effects | 471 (3.0%) | |
| Septicemia | 454 (2.9%) | |
| All other (combined) | 3,279 (20.9%) | |
| Dead (missing/unknown COD) | 1,784 (1.3%) | |
| N/A not first tumor | 2,839 (2.0%) | |
| DC- Death certificate; COD- Cause of death  141,651 (70%) patients died during follow-up. | | |
